# TERT promoter mutations in renal cell carcinomas and upper tract urothelial carcinomas

**DOI:** 10.18632/oncotarget.1829

**Published:** 2014-03-16

**Authors:** Kun Wang, Tiantian Liu, Li Liu, Jikai Liu, Cheng Liu, Chang Wang, Nan Ge, Hongbo Ren, Keqiang Yan, Sanyuan Hu, Magnus Björkholm, Yidong Fan, Dawei Xu

**Affiliations:** ^1^ Department of Urology, Shandong University Qilu Hospital, Jinan, China; ^2^ Department of Medicine, Division of Hematology and Centre for Molecular Medicine, Karolinska University Hospital Solna and Karolinska Institutet, Stockholm, Sweden; ^3^ Department of Microbiology, Shandong University School of Medicine, Jinan, China; ^4^ Shandong University School of Nursing, Jinan, China; ^5^ Department of Urology and Central Research Laboratory, Shandong University Second Hospital, Jinan, China; ^6^ Department of General Surgery, Shandong University Qilu Hospital, Jinan, China

**Keywords:** Promoter mutation, RCC, TERT, Telomerase, UTUC

## Abstract

TERT promoter mutations are identified in many malignancies including bladder cancer (BC) and upper tract urothelial carcinoma (UTUC). In contrast, no mutations were found in renal cell carcinoma (RCC) as reported in a recent study. Because the mutant TERT promoter in urine DNA was recently tested as a marker for BC, it is important to ascertain whether these mutations are truly absent in RCCs. Here we determined TERT promoter mutations in 109 patients with RCC and 14 patients with UTUC. The mutations were found in 9/96 (9.3%) clear cell RCC (ccRCC) tumors and 1/8 (13%) chromophobe RCC tumors. Among ccRCC patients, the mutation was correlated with the advanced stages and metastasis, and higher TERT expression. Among UTUCs, the mutation was detected in tumors from 3/5 (60%) patients with renal pelvic cancer and 1/9 (11%) patients with ureter cancer. The mutation was also detected in 1 of 4 urine samples from patients with mutation+ UTUC. Collectively, TERT promoter mutations do occur in RCCs and are associated with aggressive disease. The mutation is more frequent in renal pelvic cancer. Thus, the mutant TERT promoter found in urine may come from not only BC, but also RCC or UTUC.

## INTRODUCTION

Human chromosomes terminate with 10 - 15 kb TTAGGG repetitive telomere sequences essential for chromosome stability/integrity [1-3]. Telomeric DNA is synthesized by telomerase, an RNA-dependent DNA polymerase [2,3]. Normal human differentiated cells exhibit a progressive telomere shortening with each cell division due to the lack of telomerase activity. Very short telomeres are unable to maintain their functional structure, which mimics double-strand DNA breaks, thereby activating the DNA damage response pathway and inducing cell apoptosis or senescence [1-3]. Therefore, telomere erosion prevents infinite cellular proliferation. In contrast, unlimited proliferation is a hallmark of malignant cells [4]. During the carcinogenic process, maintenance of telomere length is required for an indefinite proliferation potential, and is achieved by telomerase activation in the majority of human malignancies [1-3]. The core telomerase enzyme consists of only two components: the ubiquitously expressed RNA template and telomerase reverse transcriptase (TERT), a catalytic rate-limiting subunit. As the transcriptional activation of the *TERT* gene is intimately coupled with the acquisition of telomerase activity in transformed cells, the regulatory mechanism controlling the TERT transcription has been extensively investigated in recent years. However, it remains incompletely understood how the cancer-specific TERT transcription is activated [2,3]. Recently, TERT promoter mutations have been reported in human malignancies, which create *de novo* ETS1 binding motifs (gain of function), thereby facilitating the TERT transcription [5,6]. This genetic event is thus a novel mechanism activating telomerase in malignant cells. The two recurrent mutations C228T and C250T were first described in melanoma [5,6], and have since then been detected in a variety of human tumors [7-19]. Clinical observations suggest that TERT promoter mutations may predict outcomes and also serve as markers of diagnosis or recurrence in a number of cancer types [7-9,14-18]. For instance, up to 80% of patients with bladder cancer (BC) carry TERT promoter mutations in their tumors, and detection of these mutations in urine has recently been tested at both diagnostic work-up and monitoring of recurrence [15-18]. The preliminary results look promising.

As urine-based tests are also applied for other urological malignancies including renal cell carcinoma (RCC), and upper tract urothelial carcinomas (UTUC), it is important to ascertain whether TERT promoter mutations are specific to BC only or widely present in different urological malignancies. RCC, as one of the most common urological tumors, is a heterogeneous group of malignancies derived from the epithelium of the renal tubules and consists predominantly of clear cell RCC (ccRCC, up to 80%), papillary RCC (pRCC, 10%) and chromophobe RCC (chRCC, 5%) [20-22]. UTUC is less frequent and comprises approximately 5% of urothelial carcinomas [23]. Like other human malignancies, most RCCs and UTUCs exhibit telomerase activation [24,25], which suggests a possibility that TERT promoter mutations occur in these tumors. However, Vinagre et al analyzed 26 RCC tumors including 12 ccRCCs, 4 chRCCs and 10 PRCCs, and did not find any mutation-positive cases [8]. In addition, a previous study showed a 50% frequency of TERT promoter mutations in UTUC, but it is unclear whether these mutations are detectable in urine from patients with UTUC, as seen in BC, and whether the mutations are clinically relevant in UTUCs. In the present study, we sought to address these issues by determining the mutational status of the *TERT* promoter in a large cohort of RCC patients and a group of UTUC patients, and to explore its associations with clinical and pathological characteristics of RCCs and UTCCs.

## RESULTS

### Identification of TERT promoter mutations in tumors from RCCs and UTUCs

**Figure 1:**
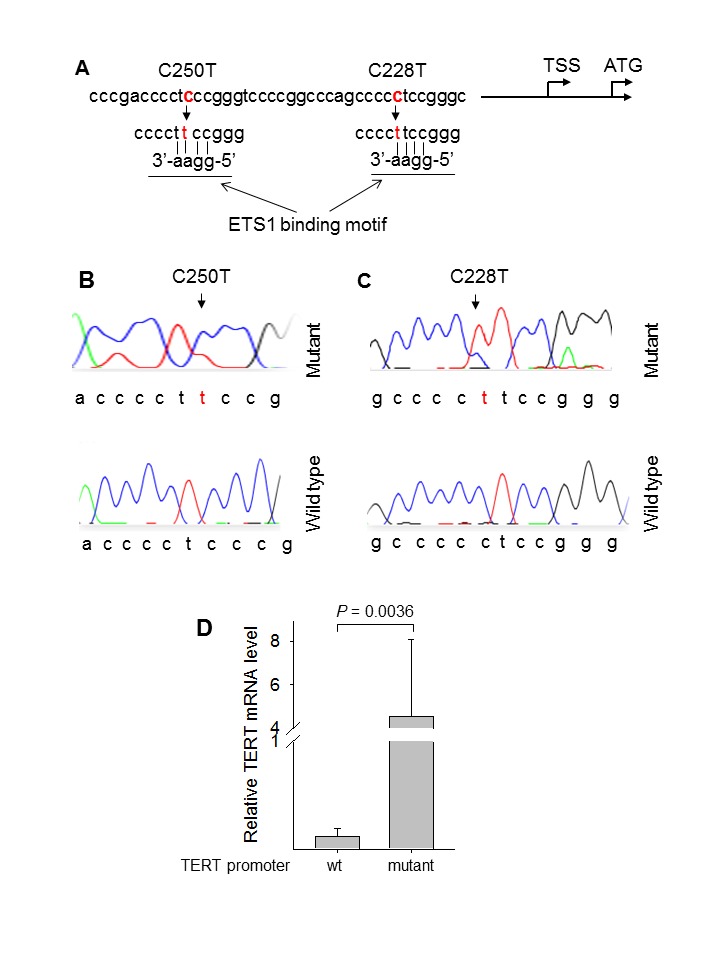
Identification of *TERT* promoter mutation in renal cell carcinomas (A) Location of C228T and C250T (in red) in the TERT core promoter. TSS: Transcription start site. The mutations create *de novo* binding motifs (GGAA) for the transcription factor ETS1. (B) and (C) Sequencing chromatographs of the *TERT* promoter locus in tumor genomic DNA from two ccRCC patients obtained by Sanger sequencing. Top panel: C to T transition at C250 (heterozygous) (B) and C228 (C) in the *TERT* promoter locus were identified in the two tumors, respectively. Of note: A heterozygous C to T transition at C254 was also observed. Bottom panel: Sequencing chromatographs of the wild type *TERT* promoter locus in two ccRCC tumors. (D) TERT mRNA expression in ccRCC tumors with and without TERT promoter mutations. Quantitative real-time PCR was used to determine TERT mRNA abundance. The TERT mRNA level was expressed arbitrarily according to the threshold cycles and normalized with β2-M as described in Materials and Methods. Column: TERT mRNA levels and bars: Standard deviation. Wt: wild type TERT promoter and mutant: TERT promoter mutations.

The tumor specimens derived from 109 patients with RCC and 14 patients with UTUC were analyzed for TERT promoter mutations. A total of 10 RCC tumors were found to harbor the mutations, among which 9 were C228T while 1 was C250T (Fig. 1A, B and C). The observed frequencies in RCCs according to histology were 9/96 (9.3%) and 1/8 (13%) for ccRCCs and chRCCs, respectively (Table 1). The C228T mutation-positive chRCC was an eosinophilic variant. The mutation was not detected in the rest types of RCC (Table 1). To determine whether the mutation was germ-line, we further analysed adjacent normal renal tissues derived from patients with TERT promoter mutation-positive RCCs and only detected wt TERT promoter sequences in those normal renal samples. It is evident from the present result that the TERT promoter mutation occurring in RCCs is somatic, as observed in other types of sporadic cancer [5,7-9,14,19].

**Table 1:** Summary of TERT promoter mutations detected in renal cell carcinoma and upper tract urothelial carcinoma

	Number	mutated	wild-type
Tumor type	investigated	number (%)	number
Renal cell carcinoma (RCC)			
Clear cell renal cell carcinoma (ccRCC)	96	9 / 96 (9.3%)	87 / 96 (90.7%)
Chromophobe renal cell carcinoma (chRCC)	8	1 / 8 (13%)	7/ 8 (87%)
Papillary renal cell carcinoma (PRCC)	2	0 / 2 (-)	2 / 2 (100%)
Carcinoma of the collecting ducts of Bellini	1	0 /1 (-)	1 / 1 (100%)
Mucinous tubular and spindle cell carcinoma	1	0 / 1 (-)	1 / 1 (100%)
Xp 11 translocation carcinoma	1	0 / 1 (-)	1 / 1 (100%)
Upper tract urothelial carcinoma (UTUC)			
Renal pelvic cancer	5	3 / 5 (60%)	2 / 5 (40%)
Ureter cancer	9	1 / 9 (11%)	8 / 9 (89%)

In 14 UTUC tumor specimens, the sequencing result revealed TERT promoter mutations in 3/5 (60%) renal pelvic cancer and 1/9 (11%) ureter cancer, and all of them were C228T mutations (Table 1 and 2).

**Table 2:** TERT promoter mutations detected in both cancer and urine samples from patients with upper tract urothelial carcinoma

Patient No.	Sex	Age (year)	Diagnosis	Size (cm)	TNM	TERT promoter	
						Tissue	Urine
1	M	64	RPC, HG, invasive	2.0	T3N0M0	C228T	C228T
2	M	64	RPC, HG, invasive	3.0	T2N0M0	C228T	wt
3	F	67	RPC, LG, invasive	1.9	T1N0M0	C228T	wt
4	M	80	RPC, HG, invasive	3.2	T3N0M0	wt	wt
5	F	73	RPC, HG, invasive	3.6	T2N0M0	wt	wt
6	M	67	UC, LG, invasive	2.1	T2N0M0	wt	wt
7	M	87	UC, LG, non-invasive	2.1	TaN0M1	C228T	wt
8	F	72	UC, HG, noninvasive	0.3	T1N0M0	wt	wt
9	F	68	UC, HG, invasive	1.2	T3N0M0	wt	wt
10	F	82	UC, HG, invasive	3.9	T3N0M0	wt	N.E
11	M	71	UC, LG, noninvasive	1.2	TaN0M1	wt	wt
12	M	61	UC, HG, invasive	1.9	T1N0M0	wt	wt
13	M	64	UC, HG, invasive	3.0	T3N0M0	wt	N.E.
14	M	67	UC, HG, invasive	2.3	T3N0M0	wt	wt

RPC, renal pelvic cancer; UC, Ureter cancer; HG, high grade, and LH, low grade. N.E., non-evaulable

### Identification of TERT promoter mutations in urine from patients with UTUC

Urine DNA from all 14 patients with UTUC was analysed for TERT promoter mutations and the result was documented in Table 2. Two urine samples were not evaluable due to very low amounts of DNA (Table 3). The C228T mutation was detected in 1 urine DNA from 4 patients whose tumors carried mutations, while a wild type TERT promoter was observed in 11 evaluable urine samples derived from 8 patients with the mutation-negative tumors and 3 patients with mutation-positive tumors (Table 3). The result suggests that the mutant TERT promoter is detectable in urine from UTUC patients, although the assay sensitivity by Sanger sequencing is low.

**Table 3:** Clinical characteristics of patients with ccRCC in relation to TERT promoter mutations

	TERT promoter mutation		
Parameter	Mutated	wild-type	P - value
informative cases (n = )	n = 9	n = 87	
Age at diagnosis (n = 95)			
Mean years	50 ± 12	55 ± 11	n.s. (0.142)
Median (range) years	48 (29-65)	55 (38-78)	n.s (0.200)
Gender (n = 96)			
Female	3	31	n.s. (0.164)
Male	6	56	
Tumor size (n = 96)		9	
< 7 cm	6	73	n.s. (0.885)
> 7 cm	3	14	
Metastases or capsular invasion (n = 96)			
Yes	5	7	0,001
No	4	80	
Stages (n = 96)			
I + II	4	78	0.003
III + IV	5	9	

n.s. = not statistically significant; Significant P-values are indicated in bold ccRCC: Clear cell renal cell carcinoma

### The positive correlation between TERT promoter mutations and TERT mRNA expression in ccRCCs

As either C228T or C250T mutation leads to the enhanced transcription of the *TERT* gene by introducing gain of function binding sites for the ETS1 transcription factor, we sought to determine whether TERT mRNA expression was correlated with its promoter mutation in ccRCC. Total cellular RNA derived from ccRCC tumors was analyzed using quantitative real-time PCR (qRT-PCR), and the result revealed a dramatic difference in levels of TERT mRNA between tumors with and without TERT promoter mutations, with significantly higher TERT expression seen in mutation-positive ccRCCs (4.55 ± 3.53 vs 0.115 ± 0.08 for mutant vs wt groups, *P* = 0.0036) (Fig. 1D).

### The association of TERT promoter mutations with clinical variables in ccRCCs and UTUCs

Age-related TERT promoter mutations have been observed in different types of malignancies, and therefore age difference was first examined between patients with and without the mutation. Unexpectedly, we observed that the ccRCC patients carrying the mutation were younger than those having a wild type TERT promoter, although the difference was not statistically significant [mutation+ vs -: 50 ±12 (mean ± 12) vs 55 ± 11, P = 0.142] (Table 3). There were no differences in gender between two groups. In addition, tumor sizes did not differ in relation to mutational status (mutation+ vs -: 5.8±2.4 vs 5.4±2.4, *P* = 0.617). When tumor sizes were classified using 7 cm as a cut-off [22], the difference remained not significant (*P* = 0.195) (Table 3). However, we observed a highly significant stage dependency (Table 3). Among 96 ccRCC patients, 5 of 9 (56%) and 9 of 87 (10%) were at clinical stage III/IV in the mutation+ and - groups, respectively (*P* = 0.003).

In UTUCs, TERT promoter mutation+ and – tumors did not show differences in age, gender, tumor sizes, and TNM stages (Table 2).

### The correlation of TERT promoter mutations with aggressive ccRCCs

We then determined a potential association between TERT promoter mutations and RCC aggressiveness. Metastasis and renal capsular invasion characterize aggressive forms of RCCs and predict a poor outcome [21,26,27]. Local or distant metastases occurred in 6 of 96 patients with ccRCCs, 4 of them had a wild type of the TERT promoter while two carried a mutant promoter. In total, 2/9 of patients with ccRCC carrying TERT promoter mutations had metastatic disease while the corresponding fraction among patients with mutation-negative tumors was only 4/87 (Table 3). Capsular invasion was observed in 3/9 (33%) and 3/86 (3%) of TERT promoter mutation+ and - ccRCC tumors, respectively (Table 3). Altogether, metastasis and capsular invasion occurred in 5/9 (56%) and 7/87 (8%) of patients with ccRCC tumors with and without TERT promoter mutations, respectively (*P* = 0.001). Thus, TERT promoter mutations are associated with aggressive ccRCCs.

### The relationship between Von Hippel-Lindau (VHL) gene alterations and TERT promoter mutations in ccRCCs

The *VHL* locus was sequenced in all ccRCC tumors and 77 of them were evaluable. We identified 21 missense point mutations, 12 nucleotide deletions and 5 insertions. Collectively, 42% of patients (32/77) had one or two kinds of alterations in the *VHL* locus, among which were 4 mutant TERT promoter -carrying tumors and 28 with a wild type TERT promoter. The frequency of the *VHL* alterations did not differ significantly between tumors with and without TERT promoter mutations (4/9, 44% vs 28/68, 41%).

## DISCUSSION

The somatic mutations of the TERT promoter have recently been identified in different types of human malignancies [5-19], and studies have also shown that these mutations may serve as a new cancer diagnostic and prognostic marker [7,9,14,17-19]. Preliminary results of TERT mutation detection in urine of patients with BC seem promising. Thus, it will be very important to ascertain whether RCC tumors are exclusively free from these mutations, as reported in a previous publication [8]. In addition, approximately 50% of UTUCs were observed to harbour TERT promoter mutations [7], but it is unclear whether these mutations are also detectable in patients' urine. We thus analyzed RCC tumors from a cohort of 109 patients, and our results demonstrate that TERT promoter mutations do occur in 9.3% (9/96) of ccRCCs and in 13% (1/8) of chRCCs, although the frequency is lower than that observed in BC. The presence of TERT promoter mutations is closely associated with aggressive or invasive ccRCCs. In UTUCs, we identified C228T mutations in 3/5 patients (60%) with renal pelvic cancer and 1/9 (11%) with ureter cancer. Moreover, the mutation could be detected in urine from those patients with the mutation-positive UTUC.

Urine-based tests are a non-invasive diagnostic tool for urological malignancies including BC, RCC, UTUC, and others. Because TERT promoter mutations have never been observed in normal human cells/tissues, they are likely ideal urinary markers for diagnosis and recurrence surveillance of above urological cancers. In retrospective, proof of principle studies on TERT promoter mutations as markers for BC, BC diagnosis was already known and its discrimination with other urological malignancies needed not to be considered. Practically, however, TERT promoter mutations occur in not only BC, but also RCC and UTUC, as demonstrated here, and therefore, complementary clinical and laboratory tests are required to distinguish between BC and RCC or UTUC when the mutant TERT promoter is detected in patients' urine.

Unexpectedly, although 95% of both renal pelvic and ureter cancers are transitional cell carcinoma [23], the former exhibits a high frequency of TERT promoter mutations, which is close to that in BC, whereas the mutation rate in the later is comparable to RCCs. It is currently unclear why there is such a big difference among these seemingly relevant carcinomas. Nevertheless, the frequent TERT promoter mutation makes it the most common genetic event in renal pelvic cancer, and may serve as a useful urinary marker in this disease. Indeed, the mutant TERT promoter sequence was detected in urine from one of 3 patients with mutation-carrying renal pelvic cancer by using Sanger sequencing. Clearly, the sensitivity of Sanger sequencing is too low and we are currently testing other approaches to improve the detection sensitivity.

Widespread telomerase activation occurs in RCCs, but the underlying mechanism is incompletely understood [24]. Both C228T and C250T mutations generate gain of function ETS1 binding motifs, and the mutant TERT promoter exhibits higher transcriptional activity than does its wild type counterpart in cancer cells, thereby contributing to cancer-specific telomerase activation [5]. Interestingly, ETS1 expression is enhanced by hypoxic inducible factors (HIFs) and significantly up-regulated in ccRCC due to the aberrant accumulation of HIFs resulting from *VHL* gene inactivation [28,29]. Conceivably, the activating mutation of the TERT promoter, when coupled with ETS1 over-expression, will exert a strong effect on telomerase activation in ccRCCs. Consistently, we did observe significantly higher levels of TERT mRNA in TERT promoter mutation-carrying ccRCC tumors than in those lacking the mutation.

TERT promoter mutations are associated with certain genetic events in several malignancies [30]. For instance, these genetic events are predominant in thyroid cancer carrying the mutation in the genes of the RAS-BRAF signalling pathway, and in glioblastoma with the mutation in the *IDH1* gene [14,30]. Because the *VHL* gene alteration plays a key role in ccRCC pathogenesis [28,29], we further investigated whether TERT promoter mutations are correlated with the genetic inactivation of VHL. Our results suggest that these two genetic events are not relevant in ccRCC.

Higher levels of TERT expression or telomerase activity promote cancer progression and serve as prognostic markers in a number of human malignancies [31-33]. Moreover, TERT has been shown to induce epithelial-mesenchymal transition and a cancer stem cell phenotype, thereby promoting cancer metastasis and invasion [34,35]. Given the observation that TERT promoter mutations contribute to enhanced TERT transcription and telomerase expression, the presence of these genetic events might be associated with a poorer prognosis. This is indeed the case in glioblastomas, thyroid cancer and certain other malignancies [7-9,14]. In the present study, we similarly observed that patients with ccRCCs carrying TERT promoter mutations had advanced and aggressive disease. However, we are at the moment unable to directly answer whether the presence of TERT promoter mutations can help to predict outcome in ccRCC patients. This will need longer follow-up of patients and possibly additional studies for validation of results.

In summary, we show here that a subset of RCC tumors do harbour TERT promoter mutations. Therefore it should be borne in mind that TERT promoter mutations may be detectable also in urine from RCC patients, as shown in patients with BC. The high frequency of TERT promoter mutations in renal pelvic cancer may also serve as a potential urinary marker for this disease. Evidently, it is important to distinguish between BC and RCC or UTUC by complementary examinations, when the presence of the mutant TERT promoter in urine has been found. In addition, we also observed that TERT promoter mutations were closely associated with aggressive RCCs, which suggests potential clinical implications. Further studies are required to evaluate its prognostic value in RCCs and diagnostic value in renal pelvic cancer.

## MATERIALS and METHODS

### Patients and tumor specimens

The study was conducted on 109 patients with RCC and 14 patients with UTUC who underwent surgery at Shandong University Qilu Hospital, China, between 2011 and 2013. RCC were diagnosed according to the criteria of the World Health Organization (WHO) [20]. The RCC patients included 96 ccRCCs, 8 chRCCs, 2 pRCCs (type II), 1 Collecting duct RCC, 1 mucinous tubular and spindle cell carcinoma and 1 X11.2 translocation RCC, according to the WHO classification of renal tumors [20]. Among 14 patients with UTUC were 5 with renal pelvic cancer and 9 ureter cancer. Information was collected concerning clinical characteristics including: gender, age at diagnosis, tumor size and other histo-pathological characteristics, stages, metastases and capsular invasion. The specimens were collected after surgical treatment and kept frozen at −70°C or paraffin-embedded until use. All samples were collected with informed consent and approval by the local ethics committee.

### Voided urine samples from patients with UTUC

Urine was collected before surgical treatment from 14 patients with UTUC. Fifty ml of urine were centrifuged and the pellet was kept at −70°C until use.

### DNA extraction and sequencing

Genomic DNA was extracted from frozen and/or paraffin-embedded tumor tissue samples, and urine pellets using QIAGEN DNA extraction kits.The two mutations defined as C228T and C250T in the TERT core promoter correspond to positions 124 and 146 bp upstream of the ATG site (Fig. 1A). The target region was amplified using PCR followed by Sanger sequencing as described [9]. The PCR was performed with the following primer pairs: 5'-CACCCGTCCTGCCCCTTCACCTT-3' (forward) and 5'-GGCTTCCCACGTGCGCAGCAGGA-3' (reverse). The C228T and C250T mutations were verified by sequencing from both directions. The same set of DNA from these specimens was also analyzed for the alterations of the *VHL* gene by Sanger sequencing and the specific PCR primers are as follow [28]: Exon 1, 5′-CGAAGACTACGGAGGTCGAC-3′ (forward) and 5′-GGCTTCAGACCGTGCTATCG-3′ (reverse); Exon 2, 5′-GGCTCTTTAACAACCTTTGC-3′(forward) and 5′-TTGGATACCGTGCCTGACATC-3′ (reverse); and Exon 3, 5′-ACAGGTAGTTGTTGGCAAAGCC-3′ (forward) and 5′-GAAGGAACCAGTCCTGTATC-3′ (reverse).

### qRT-PCR for TERT mRNA expression

Total cellular RNA was extracted using Trizol kit (Life Technology) and used for cDNA synthesis. qRT-PCR was carried out in an ABI 7900HT Real time PCR System (Applied Biosystems, Foster City, CA) using SYBR Green kit (Applied Biosystems) and the following primers: TERT, 5'-CGGAAGAGTGTCTGGAGCAA-3' (forward) and 5'-GGATGAAGCGGAGTCTGGA-3' (reverse); β2-M, 5'-GAATTGCTATGTGTCT GGGT-3' (forward) and 5'-CATCTTCAAACCTCCATGATG-3' (reverse). Levels of TERT mRNA were calculated from threshold cycle values and normalized to β2-M mRNA abundance, and expressed as arbitrary units.

### Statistical analyses

Differences in the TERT promoter mutation frequency between tumors with *VHL* gene alteration, gender, clinical stage, metastasis and renal capsular invasion were determined using Fisher´s exact test. Student's T test was used to analyze differences in TERT mRNA expression, age and tumor size between the TERT promoter mutation + and – groups, respectively. All the tests were two-tailed and computed using SigmaStat3.1® software (Systat Software, Inc., Richmond, CA). *P* values of <0.05 were considered as statistically significant.
